# Decitabine Downregulates TIGAR to Induce Apoptosis and Autophagy in Myeloid Leukemia Cells

**DOI:** 10.1155/2021/8877460

**Published:** 2021-01-18

**Authors:** Lanzhu Li, Wenjie Liu, Qian Sun, Han Zhu, Ming Hong, Sixuan Qian

**Affiliations:** Department of Hematology, The First Affiliated Hospital of Nanjing Medical University, Jiangsu Province Hospital, 300 Guangzhou Road, Nanjing, Jiangsu 210029, China

## Abstract

Decitabine (DAC) is a well-known DNA methyltransferase inhibitor, which has been widely used for the treatment of acute myeloid leukemia (AML). However, in addition to hypomethylation, DAC in AML is also involved in cell metabolism, apoptosis, and immunity. The TP53-induced glycolysis and apoptosis regulator (TIGAR) functions to inhabit glycolysis and protect cancer cells from reactive oxygen species- (ROS-) associated apoptosis. Our previous study revealed that TIGAR is highly expressed in myeloid leukemia cell lines and AML primary cells and associated with poor prognosis in adult patients with cytogenetically normal AML. In the present study, it was found that in a time- and concentration-dependent manner, DAC downregulates the TIGAR expression, induces ROS production, and promotes apoptosis in HL-60 and K562 cells. However, blocking the glycolytic pathway partially reversed the combined effects of DAC and TIGAR knockdown on apoptosis, ROS production, and cell cycle arrest, indicating that DAC induced apoptosis through the glycolytic pathway. Furthermore, TIGAR also has a negative impact on autophagy, while DAC treatment upregulates autophagy-related proteins LC3, Beclin-1, ATG3, and ATG-5, downregulates p62, and promotes the formation of autophagosomes, indicating that DAC may activate autophagy by downregulating TIGAR. Taken together, DAC plays an unmethylated role in inducing apoptosis and activating autophagy in myeloid leukemia by downregulating TIGAR.

## 1. Introduction

Leukemia is a malignant clonal disease of hematopoietic stem cells. Myeloid leukemia, including acute myeloid leukemia (AML) and chronic myeloid leukemia (CML), is the most common malignant disease of the blood system. Its pathogenesis is complex. In addition to the cytogenetic changes, many epigenetic modifications have also been found. The 5-aza-2′-deoxycytidine (decitabine, DAC) is an FDA-approved DNA methyltransferase (DNMT) inhibitor used for the treatment of myelodysplastic syndromes (MDS) and AML [1], and this has been recently considered in the treatment of solid tumors [2]. DAC in combination with other chemotherapeutic drugs has been proven to have additive or synergistic biological activity, which may reverse clinical drug resistance [3, 4]. However, the data that correlates DNA methylation reversal and clinical response have been conflicting, and the actual mechanism of DAC on leukemic cells is much more complex than previously considered. In recent years, the DAC nonmethylation mechanism that induced the apoptosis of leukemia cells has attracted increasing attention. It was found that DAC has antimetabolic effects on a variety of cancer cells [5, 6]. Furthermore, it has been reported that DAC induces the reactive oxygen species- (ROS-) associated apoptosis of leukemia cells [7, 8] and can induce autophagy in cancer cells [9, 10]. Our previous study suggested that myeloid leukemia cells have abnormal energy metabolism [11], and that TIGAR (TP53 inducing glycolysis and apoptosis regulator) is highly expressed in myeloid leukemia cell lines and AML primary cells and associated with poor prognosis in adult patients with cytogenetically normal AML, which can be inhibited by DAC [12]. Therefore, it was speculated that DAC may play an important role in nonmethylation in myeloid leukemia by downregulating the TIGAR gene.

The concept of the Warburg effect describes that cancer cells preferentially utilize the glycolytic pathway to produce ATP even in the presence of oxygen. For instance, lung cancer, which is always exposed to hyperoxygen [13] and leukemic cells in the bloodstream [14], have been shown to have a high degree of aerobic glycolysis during tumor formation. TIGAR, which is a metabolic enzyme, functions to lower the fructose-2,6-diphosphate (Fru-2,6-P_2_) levels in cells, converting metabolism to the pentose phosphate pathway (PPP). This would result in the increase in nicotinamide adenine dinucleotide phosphate (NADPH) and glutathione (GSH) and the decrease in ROS levels [15]. ROS can oxidize and destroy the mitochondria of eukaryotic cells, leading to mitochondrial dysfunction and cell damage [16]. Therefore, TIGAR protects cells from damage by affecting the glycolysis and regulating intracellular NADPH and ROS levels, thereby promoting cell growth and proliferation and inhibiting apoptosis and autophagy. Since the function of TIGAR is closely correlated to regulate the cellular redox status, this was a great significance to the study of the expression of TIGAR and its related effects due to DAC treatment in human myeloid leukemia cell lines.

In the present study, the investigators observed that DAC downregulated the expression of TIGAR and induced ROS production, which led to the cell apoptosis of HL-60 and K562 cells. In addition, DAC may activate autophagy by downregulating TIGAR. These data provide evidence that TIGAR may be a potential therapeutic target in the treatment of leukemia with DAC.

## 2. Material and Methods

### 2.1. Reagents

Decitabine supplied by Sigma (St. Louis, Mo, USA) was dissolved in dimethyl sulfoxide (DMSO) (Invitrogen, USA) at a 20 mM concentration for stock solutions. The stock solutions were stored at -80°C. Imatinib was purchased from Novartis (Beijing, China) and also dissolved in DMSO at a 20 mM concentration and kept at -80°C. The stock solutions were diluted to working concentrations in the subsequent experiments with growth media. Control groups were treated with an equal amount of DMSO (<0.1%) in the corresponding experiments: 1 mg/ml 2-deoxy-D-glucose (2-DG) (Sigma-Aldrich, USA) for 48 h to suppress glycolysis. The primary antibodies against TIGAR (E11-202999), caspase-3 (E2510665), caspase-8 (E2310134), caspase-9 (E300015), cytochrome c (E11-0170C), ATG5 (E2600557), ATG3 (E306919), LC3 (E2508295), and Beclin-1 (E2612473) were from EnoGene (Nanjing, China). Anti-Bcr-abl (ab187831) and anti-p-Tyr-100 (ab129991) were from Abcam (Cambridge, UK).

### 2.2. Cell Culture

Human leukemia HL-60 (acute promyelocytic leukemia cell lines) and K562 (chronic myeloid leukemia in blast crisis) cells were obtained from the American Type Culture Collection, and K562/R (Imatinib resistant K562 cells) cells were obtain from OGpharma (Nanjing, China) and cultured in RPMI-1640 medium (GIBCO, USA) containing 10% fetal bovine serum, 2 mM of L-glutamine, 50 U/ml of penicillin, and 50 *μ*g/ml of streptomycin in a humidified incubator at 37°C under 5% CO_2_ atmosphere.

### 2.3. Cellular Transfection and RNA Interference

Small interfering RNAs (siRNAs) and the overexpression plasmid were purchased from the GenePharma Corporation (Shanghai, China) and GeneChem (Shanghai, China), respectively. Lipofectamine 2000 (Invitrogen, USA) was used to transfect the siRNA or plasmid, according to manufacturer's instructions. Further analysis and experiments were carried out at 48 hours after transfection. The sequence of siRNAs was as follows: siTIGAR, 5′-TTAGCAGCCAGTGTCTTAG-3′; NC, 5′-TTACCGAGACCGTACGTAT-3′. The construction of the plasmid was pSRL-SIH1-H1-Puro.

### 2.4. ROS and Apoptosis Assay

The DCFH-DA probe (S0033; Beyotime Institute of Biotechnology, Haimen, China) was diluted in serum-free medium at 1 : 1,000 to achieve a final concentration of 10 *μ*mol/L. Then, the HL-60 and K562 cells were collected by centrifugation, and the cell culture medium was removed. Afterwards, 500 *μ*l of diluted DCFH-DA was added and incubated at 37°C for 30 minutes, with shaking every five minutes. Next, cells were washed with PBS (three times, for five minutes each time) to remove the extracellular residual DCFH-DA probes. In order to quantify the ROS levels, the mean fluorescence intensity (MFI) of cells was analyzed by flow cytometry (FACS Calibur, BD Biosciences).

The apoptosis assay was performed using an ApoScreen Annexin V Apoptosis Kit (Southern Biotech Cat. No. 10010-09), according to manufacturer's instructions. Briefly, cells were collected and washed for three times with PBS before being counted and resuspended (2 × 10^5^) in 200 *μ*l of binding buffer. Then, 10 *μ*l of Annexin-V-R-PE and 7-AAD were added to the cell suspensions, and the sample was incubated in the dark for 15 minutes. Afterwards, the apoptotic cells were detected by flow cytometry within one hour.

### 2.5. Nicotinamide Adenine Dinucleotide Phosphate (NADPH) Estimation

After the indicated treatment, cells were washed with PBS and collected. Then, the NADPH concentrations were measured using the NADPH detection kit obtained from the Biyuntian Biotechnology (Shanghai, China), according to manufacturer's instructions.

### 2.6. Assessment of the Cell Cycle Using PI Staining

The cell cycle distribution of HL-60 cells was measured using a PI staining assay kit (Beyotime Biotech), according to manufacturer's instructions. Briefly, cells in the logarithmic growth phase were collected by centrifugation at 1,000 r/min for 10 minutes and washed once with PBS to remove the serum. Then, the cells were counted, the concentration was adjusted to 2 × 10^6^ cells/tube with 75% ethanol, and these were fixed at -20°C for more than 24 hours. Afterwards, these cells were seeded in a 6-well plate with 2 ml of cells in each well and cultured for 24 hours. Subsequently, these cells were pelleted by centrifugation at 1,000 r/min for 10 minutes, washed once with cold PBS, and resuspended in 300 *μ*l of PBS. Next, 50 *μ*L of RNase (2.5 mg/ml) was added to each tube to reach a final concentration of 250 *μ*g/ml, mixed, and incubated at 4°C for 30 minutes. Next, 50 *μ*L of PI (100 *μ*g/ml of the final concentration) was added to the above cell suspension, mixed, and incubated in the dark at 4°C for 30 minutes. Then, the cell cycle distribution was determined by flow cytometry within one hour using a flow cytometer (FACS Calibur, BD Biosciences).

### 2.7. Phosphorylated-Tyrosine (p-Tyr) and BCR-ABL Protein Assay

K562, K562/R, and K562/TIGAR cells were collected by centrifugation at 2,000 r/min for five minutes, washed once with PBS to remove the serum, and resuspended in 100 *μ*l of PBS. Subsequently, these cells were stained using anti-Bcr-abl (1 : 100) or anti-p-Tyr-100 (1 : 50) antibodies and incubated at 37°C for one hour. After washing with PBS, these cells were resuspended with PBS containing 2% fetal bovine serum, precooled at 4°C, and submitted for flow cytometric analysis (BD Biosciences).

### 2.8. Transmission Electron Microscopy

After the indicated treatment, HL-60 cells were harvested and centrifuged at 1,000 rpm for 10 minutes. Then, cells were fixed with 2.5% glutaraldehyde for two hours and washed with PBS for three times, each time for 15 minutes. Afterwards, these were fixed with 1% osmium tetroxide for three hours and washed with PBS for three times, each time for 15 minutes. Next, these cells were dehydrated in a graded concentration of ethanol, saturated in graded acetone, cut into 50 nm ultrathin sections, stained with lead citrate, and viewed using JEM-1010 TEM (JEOL, Japan).

### 2.9. Western Blot Analysis

The total protein of human leukemia cells was extracted. Then, cells were resolved by 10% SDS-PAGE and transferred onto polyvinylidene fluoride (PVDF) membranes. After incubating in 5% skim milk for two hours, this was incubated with the corresponding primary antibody overnight at 4°C. Then, the secondary antibody was used to react with the primary antibody for 1.5 hours. Afterwards, the membrane was placed in TBST solution, shaken and rinsed for five minutes for four times, and placed in the Western Lightning™ Chemiluminescence Reagent for 30-60 seconds. Subsequently, the film was immediately placed in the exposure box, and the photosensitive film was exposed for 10 seconds in the dark, followed by visualization with ECL Plus (Millipore, Billerica, MA, USA) and the Bio-Imaging System.

### 2.10. Quantitative Real-Time Reverse Transcription PCR

Total RNA was extracted from human leukemia cells using TRIzol reagent (Invitrogen). The purity of the RNA was determined by the ratio of A 260 to A 280. The instructions in the RevertAidTM First Strand cDNA Synthesis Kit (Fermentas) was followed to synthesize the cDNA. The mRNA expression was detected by real-time PCR (RT-PCR) using the SYBR green PCR kit (Application Biosystems, Foster City, CA, USA). The RT-PCR conditions were as follows: one cycle at 94°C for 10 minutes, 40 cycles at 94°C for 10 seconds, 60°C for 30 seconds, and one cycle at 72°C for three minutes. Next, the mRNA levels of each gene were calculated through the normalization of the GAPDH protein, and the 2*ΔΔ*Ct method was applied for the analysis of the target gene expression levels. Each sample was measured in triplicate. The primer sequences used for qRT-PCR are listed in Table S1.

### 2.11. Statistical Analysis

The experimental data were expressed as the mean ± standard deviation of three independent experiments. The statistical analysis was performed using paired Student's *t*-test. *P* < 0.05 was considered statistically significant.

## 3. Results

### 3.1. DAC Inhibits the TIGAR mRNA and Protein Expression

**Figure 1:**
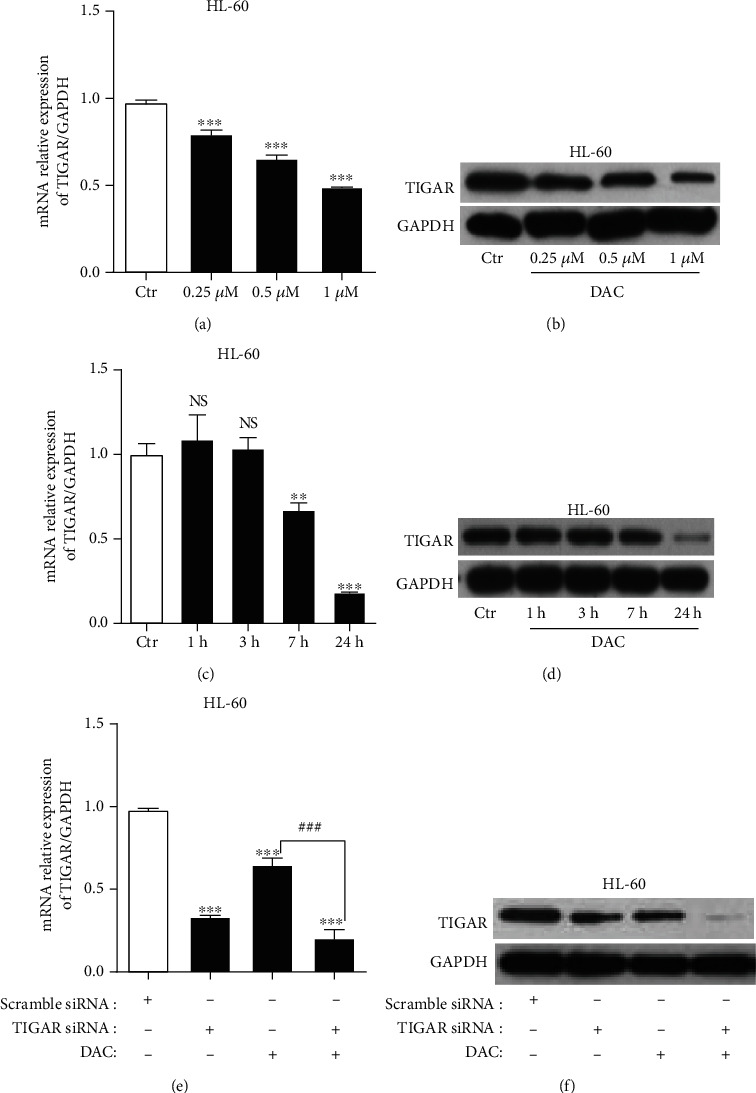
DAC inhibits the TIGAR mRNA and protein expression. The TIGAR mRNA (a) and protein (b) levels were observed when HL-60 cells were treated with different concentrations of DAC (0.25, 0.50, and 1.00 *μ*M) for 24 hours. The TIGAR mRNA (c) and protein (d) levels were observed when HL-60 cells were treated with 0.5 *μ*M of DAC for different lengths of time (1, 3, 7, and 24 hours). The TIGAR mRNA (e) and protein (f) levels were observed when HL-60 cells were treated with or without 0.5 *μ*M of DAC for 24 hours after TIGAR with or without knockdown. The values were presented as the mean ± standard deviation (SD) of three independent experiments. ^∗∗^*P* < 0.01, ^∗∗∗^*P* < 0.001, NS *P* > 0.05 compared to the control group; ^###^*P* < 0.001 compared to DAC alone.

The previous study conducted by the investigators revealed that TIGAR is highly expressed in myeloid leukemia cell lines and AML primary cells and is associated with the poor prognosis of adult patients with cytogenetically normal AML [12]. In order to detect the TIGAR expression in response to DAC treatment, the HL-60 and K562 cells were treated with different concentrations of DAC (0.25, 0.50, and 1.00 *μΜ*) for 24 hours or with 0.5 *μΜ* of DAC for different lengths of time (1, 3, 7, and 24 hours). Both the protein and mRNA levels of TIGAR decreased in a dose- and time-dependent manner after DAC treatment (Figures 1(a)–1(d)). Similar results were obtained for K562 cells (Supplementary Fig 1a-1d). In addition, the DAC treatment combined with TIGAR knockdown further reduced the TIGAR protein and mRNA levels in HL-60 cells (Figures 1(e) and 1(f)).

### 3.2. DAC Induces Intracellular ROS Production by Downregulating TIGAR

**Figure 2:**
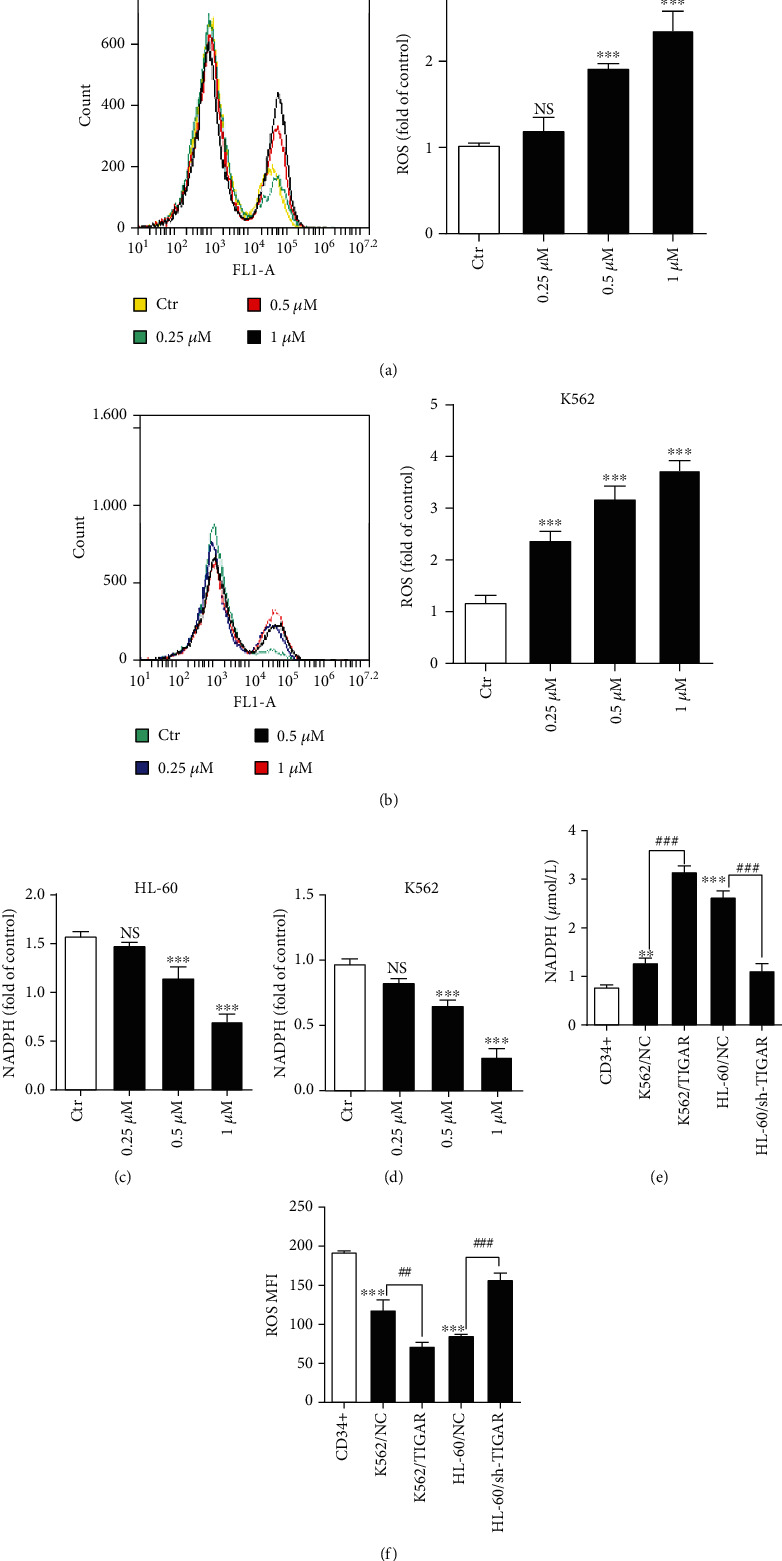
DAC induces intracellular ROS production by downregulating TIGAR. HL-60 and K562 cells were treated with different doses of DAC for 24 hours. (a, b) The production of ROS was assessed by FCM. (c, d) NADPH levels were detected by the NADPH detection kit. The NADPH (e) and ROS (f) levels in K562 cells, with or without TIGAR overexpression, and HL-60 cells, with or without TIGAR knockdown, when compared with CD34+ cells. The values were presented as the mean ± standard deviation (SD) of three independent experiments. ^∗∗^*P* < 0.01, ^∗∗∗^*P* < 0.001, NS *P* > 0.05 compared to the control group.

It has been reported that ROS is closely associated with DAC-induced apoptosis in leukemia cells [7]. Therefore, the investigators determined whether DAC induced the ROS generation in HL-60 and K562 cells using DCFH-DA. The ROS generation increased after the supplementation with different concentrations of DAC (0.25, 0.50, and 1.00 *μΜ*) for 24 hours in HL-60 and K562 cells (Figures 2(a) and 2(b)). Then, after the treatment with DAC, NADPH levels were also detected, and results revealed that the level of NADPH decreased in a concentration-dependent manner (Figures 2(c) and 2(d)). We have previously reported that TIGAR was highly expressed in several myeloid leukemia cell lines (HL-60, K562, Jurkat, and NB-4), especially in HL-60 cells, but relatively low in K562 cells [12]. In order to verify whether the increase in the ROS level induced by DAC due to the downregulation of the TIGAR expression, the investigators tested the effect of TIGAR knockdown and overexpression in HL-60 and k562 cells, respectively. The results revealed that the knockdown of TIGAR reduced the NADPH levels and increased the ROS levels in HL-60 cells. In contrast, the overexpression of TIGAR increased the NADPH levels and reduced the ROS levels in K562 cells (Figures 2(e) and 2(f)). Therefore, the increase in ROS due to DAC treatment may be the result of the decrease in the expression level of TIGAR.

### 3.3. DAC Triggered the ROS-Associated Apoptosis through the Downregulation of TIGAR

**Figure 3:**
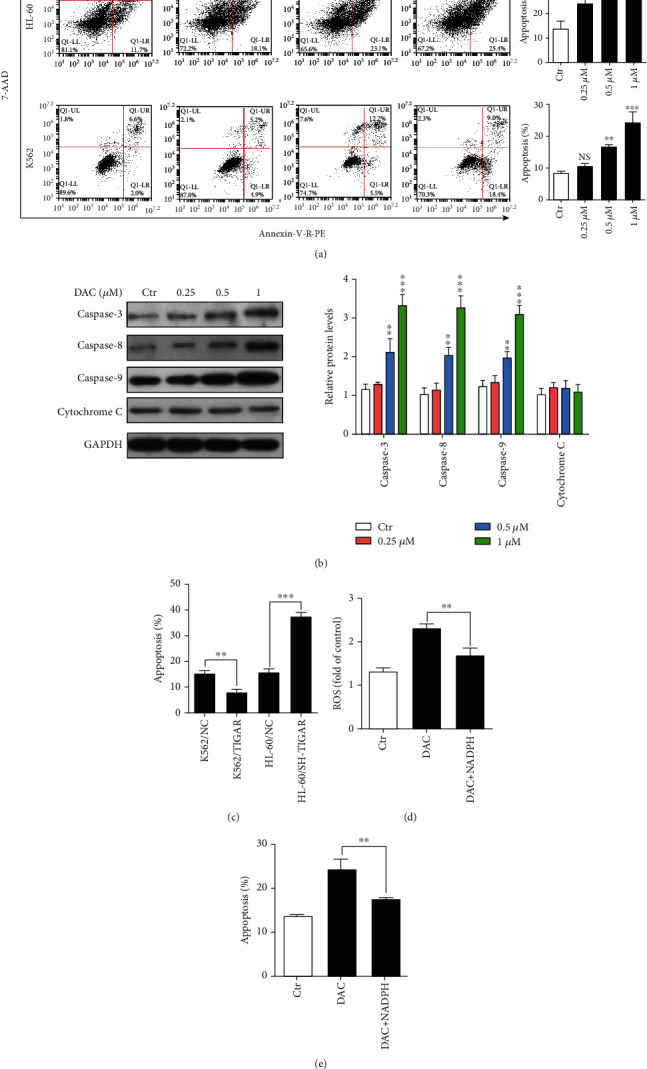
DAC triggered the ROS-associated apoptosis by downregulating TIGAR. (a) FCM analysis of the apoptosis using the double staining of Annexin-V-R-PE and 7-AAD in HL-60 and K562 cells with the treatment of 0.25, 0.50, and 1.00 *μ*M of DAC for 24 hours. (b) The levels of caspase-3, -8, and -9, and cytochrome c were detected by western blot. (c) The FCM analysis of the apoptosis of K562 cells, with or without TIGAR overexpression, and HL-60 cells, with or without TIGAR knockdown. The ROS levels (d) and apoptosis (e) of HL-60 cells after DAC treatment in the presence or absence of NADPH. The values were presented as the mean ± standard deviation (SD) of three independent experiments. ^∗∗^*P* < 0.01, ^∗∗∗^*P* < 0.001, NS *P* > 0.05 compared to the control group.

In order to investigate the effect of DAC on HL-60 and K562 cell apoptosis, two well-established assays were subsequently used for the assessment of apoptosis. First, the 7-AAD and Annexin-V-R-PE staining of HL-60 and K562 cells was performed. After the treatment with DAC (0.25, 0.50, and 1.00 *μΜ*) for 24 hours, the apoptotic percentages were 24.27 ± 2.69%, 31.07 ± 1.82%, and 30.77 ± 1.48%, respectively, in HL-60 cells and 10.63 ± 0.99%, 16.77 ± 0.83%, and 24.33 ± 3.51% in K562 cells, respectively (Figure 3(a)). Second, the investigators analyzed the protein level of caspase-3, -8, and -9 and cytochrome c after DAC treatment, which is another hallmark of apoptosis. As shown in Figure 3(b), DAC significantly increased the levels of caspase-3, -8, and -9 in HL-60 cells. However, there was no significant change in the level of cytochrome c. The overexpression and knockdown TIGAR significantly inhibited and promoted the apoptosis, respectively (Figure 3(c)). In order to further determine whether the generation of ROS is a crucial event required for the apoptotic pathway activation in response to DAC treatment, NADPH (a byproduct of the PPP, and the main ROS scavenger in cells) was supplemented to HL-60 cells. The exogenous addition of NADPH significantly decreased the level of ROS in cells treated with DAC (Figure 3(d)). The apoptosis of HL-60 cells with the treatment of DAC was attenuated by the addition of NADPH (Figure 3(e)). However, the apoptosis rate was still higher than that of the control group (*P* < 0.05), suggesting that the enhancement of the apoptosis rate by DAC treatment is partially mediated by the increase in ROS levels.

### 3.4. DAC Induces Apoptosis by Reducing p-Tyr and BCR-ABL

**Figure 4:**
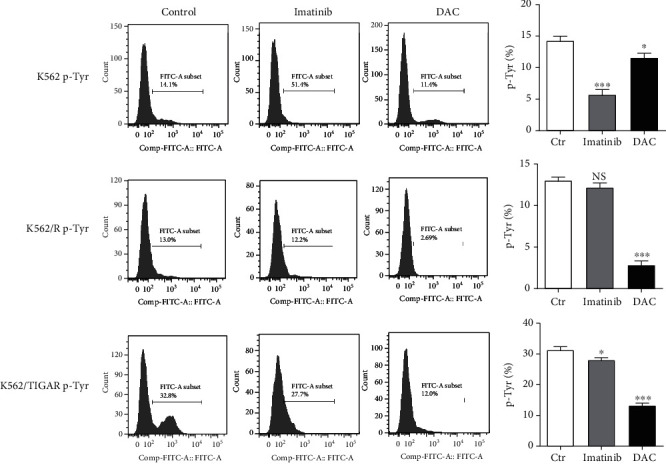
DAC induces apoptosis by reducing p-Tyr and BCR-ABL. The 0.5 *μ*M of DAC and 0.1 *μ*M of Imatinib treatment of K562, K562/R, and K562/TIGAR cells is shown, and the level of p-Tyr was assessed by FCM. The values were presented as the mean ± standard deviation (SD) of three independent experiments. ^∗^*P* < 0.05, ^∗∗∗^*P* < 0.001, NS *P* > 0.05 compared to the control group.

**Figure 5:**
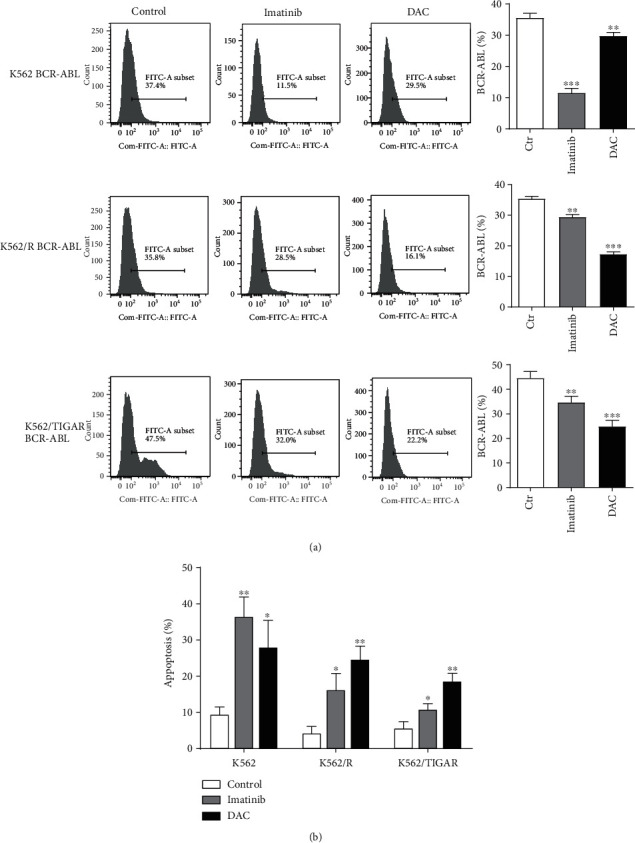
DAC induces the apoptosis by reducing p-Tyr and BCR-ABL. The 0.5 *μ*M of DAC and 0.1 *μ*M of imatinib treatment of K562, K562/R, and K562/TIGAR cells is shown, and the BCR-ABL protein (a) and apoptosis (b) were assessed by FCM. The values were presented as the mean ± standard deviation (SD) of three independent experiments. ^∗^*P* < 0.05, ^∗∗^*P* < 0.01, ^∗∗∗^*P* < 0.001 compared to the control group.

It was shown that DAC can induce the apoptosis of K562 cells (derived from the CML patient in blast crisis). BCR-ABL is an antiapoptotic protein closely correlated to the pathogenesis of CML. It was hypothesized that DAC may also induce apoptosis by downregulating the protein expression level of BCR-ABL. Imatinib was compared with DAC in the treatment of K562 cells, as well as in imatinib-resistant K562 cells (K562/R) and overexpressing TIGAR K562 cells (K562/TIGAR), respectively. Flow cytometry was performed to detect the effects of imatinib and DAC treatment on phosphorylated tyrosine kinase (p-Tyr), BCR-ABL, and apoptosis. It was found that DAC significantly inhibited the expression of p-Tyr and BCR-ABL. The inhibitory effect of DAC on K562/R and K562/TIGAR cells for p-Tyr and BCR-ABL was significantly stronger, when compared to imatinib (Figures 4 and 5(a)). Similarly, the DAC-induced apoptosis in K562/R and K562/TIGAR cells was stronger than imatinib and when compared with K562 cells (Figure 5(b)).

### 3.5. The Inhibition of the Glycolysis Pathway Attenuates the Effects of DAC

**Figure 6:**
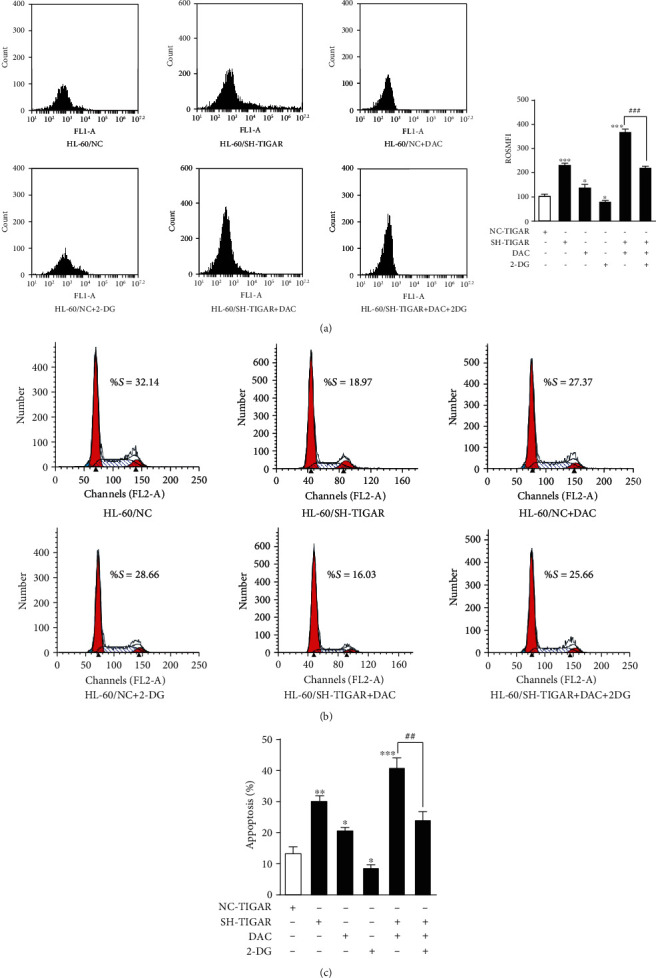
The inhibition of the glycolysis pathway attenuates the effects of DAC. The ROS levels (a), cell cycle distribution (b), and apoptosis (c) of HL-60 cells detected by FCM after TIGAR knockdown, and 0.5 *μ*M of DAC treatment, in the presence or absence of 1 mg/ml of 2-DG are shown. The values were presented as the mean ± standard deviation (SD) of three independent experiments. ^∗^*P* < 0.05, ^∗∗^*P* < 0.01, ^∗∗∗^*P* < 0.001, NS *P* > 0.05 compared to the control group.

It is well known that TIGAR slows down the glycolysis pathway. Furthermore, it was shown that DAC can downregulate the TIGAR expression in a time- and dose-dependent manner. Next, the investigators determined whether the DAC-induced apoptosis of leukemic cells was influenced by the glycolytic pathway. HL-60 cells transfected with the siRNA were treated with the glycolysis inhibitor (2-deoxy-D-glucose: 2-DG) for two hours before the treatment with DAC. Flow cytometry was employed to assess the changes in intracellular ROS, cell cycle distribution, and apoptosis rate. Through cell cycle analysis, it was found that the DAC treatment and TIGAR knockdown significantly inhibit the S-phase DNA synthesis of HL-60 cells (Figure 6(b)). However, the level of ROS, cell cycle arrest, and apoptosis rate in HL-60 cells after TIGAR knockdown with the treatment of DAC was significantly attenuated after the pretreatment with 2-DG (Figure 6). This suggests that the antileukemic effect of DAC may be correlated to the downregulation of TIGAR to promote glycolysis.

### 3.6. TIGAR Knockdown Enhanced the DAC-Induced Autophagy Activation

**Figure 7:**
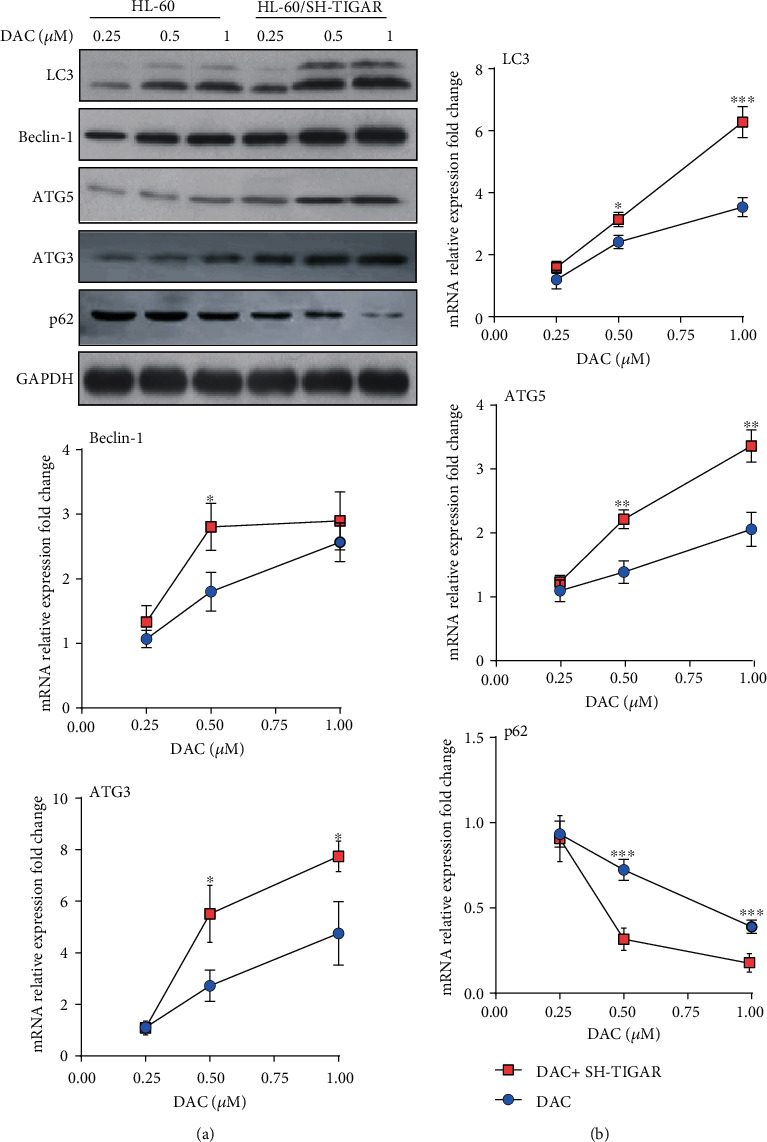
The TIGAR knockdown enhanced the DAC-induced autophagy activation. HL-60 cells were treated with 0.25, 0.50, and 1.00 *μ*M of DAC for 24 hours, with or without TIGAR knockdown. The levels of LC3, Beclin-1, and ATG5 were detected by western blot (a) and qPCR (b). The values were presented as the mean ± standard deviation (SD) of three independent experiments. ^∗^*P* < 0.05, ^∗∗^*P* < 0.01, ^∗∗∗^*P* < 0.001 compared to the control group.

**Figure 8:**
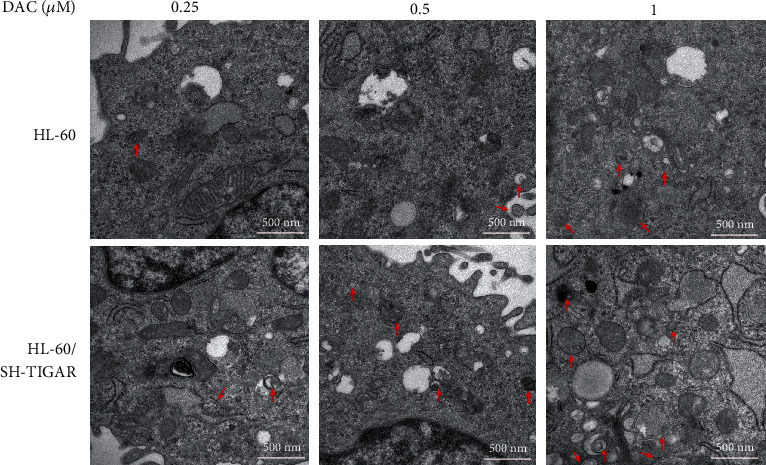
The TIGAR knockdown enhanced the DAC-induced autophagy activation. HL-60 cells were treated with 0.25, 0.50, and 1.00 *μ*M of DAC for 24 hours, with or without TIGAR knockdown. The autophagosome formation was detected by transmission electron microscopy. Scale bar: 500 nm. Red arrows indicate autophagosomes.

DAC treatment induced the production of ROS, which is a key upstream signal to activate autophagy [17]. Next, the effects of DAC on autophagy were investigated. After treating HL-60 cells with 0.25, 0.50, and 1.00 *μΜ* of DAC for 24 hours, western blot and qPCR were performed to detect the expression level of the autophagy protein. The results revealed that DAC significantly increased both the protein and mRNA levels of LC3, Beclin-1, ATG3, and ATG5 (Figures 7(a) and 7(b)) and decreased both the protein and mRNA levels of p62, indicating that DAC activated the autophagy activity. In addition, TIGAR knockdown also induced the autophagy activation, and when combined with the DAC treatment, this produced a synergistic effect on the autophagy activation (Figures 7(a) and 7(b)). In order to further confirm whether the DAC treatment and TIGAR knockdown can enhance the autophagy activity, the investigators examined the formation of autophagosomes by electron microscopy. The results revealed that with the increase in DAC concentration, the number of autophagosomes and secondary lysosomes increased in HL-60 cells (Figure 8).

## 4. Discussion

DAC is an FDA-approved DNMT inhibitor for the treatment of MDS and AML. The affirmation of clinical efficacy has led researchers to carry out many studies on the mechanism of DAC methylation [18, 19]. However, subsequent studies have revealed that the clinical efficacy of DAC in leukemia is not directly proportional to the degree of DNA methylation reduction. Oki et al. treated 28 imatinib resistance or progressive CML patients with imatinib combined with DAC, and at the same time, detected the long interspersed nucleotide element (LINE) methylation to observe the genome-wide DNA methylation levels [20]. Contrary to their expectations, the decrease in LINE methylation was more obvious in ineffective treatment patients, and it was speculated that there must be a nonmethylation mechanism in DAC in vivo to induce the apoptosis of leukemia cells [20]. In recent years, more attention has been given to its antitumor effect through its mechanism, other than the DNMT inhibition. In particular, the DAC induction of ROS production that mediated the apoptosis is a very interesting and promising antitumor property [7, 8]. The study on how DAC induces ROS-associated apoptosis in leukemia cells is of great significance for understanding the antitumor effect of DAC. In the present study, it was found that TIGAR (an important regulator of oxidative stress) is the target of DAC-induced ROS production to mediate leukemia cell apoptosis.

The TIGAR protein is known to shift the glycolytic metabolism to PPP, which assists in the generation of NADPH to maintain the levels of GSH and thereby reduce the intracellular ROS. In the present study, it was further confirmed that TIGAR can increase the level of NADPH and decrease the level of ROS in myeloid leukemia cells by silencing or overexpressing TIGAR in HL-60 and K562 cells, respectively (Figures 2(e) and 2(f)). These results revealed that DAC downregulated the expression of the TIGAR protein and gene in HL-60 and K562 cells in a time- and concentration-dependent manner (Figures 1(a)–1(d), Supplementary Fig 1a-1d). Thus, this alleviated the inhibitory effect of TIGAR on ROS production, resulting in a significant increase in the intracellular ROS level (Figures 2(a) and 2(b)). ROS have been generally regarded as byproducts of oxygen consumption and cell metabolism. At normal concentrations, ROS serves as important second messengers involved in various signal transduction events that regulate cell differentiation, proliferation, or apoptosis [21, 22]. However, excessive concentrations of ROS can lead to oxidative damage to cells and tissues, which is associated with the development of cancer and many other human diseases [23, 24]. ROS are the most effective activators of signal-regulating kinase 1 (ASK1). After the ASK1 signal body is activated, this activates the downstream effector mitogen-activated protein kinases (MAPKs), such as J Jun N-terminal protein kinase (JNK). JNK further activates the downstream proapoptotic molecules and/or inhibits the antiapoptotic molecules and their regulators to promote apoptosis [25]. In order to understand the role of ROS in DAC-induced apoptosis, the replenishment of NADPH (a byproduct of the PPP, and the main ROS scavenger in cells) were performed to partly rescue the apoptosis rate of HL-60 cells after DAC treatment (Figure 3(d)), indicating that ROS acts as upstream signaling molecules for the initiation of cell apoptosis. In addition, the synergistic effects of DAC combined with TIGAR knockdown on ROS production, cell cycle arrest, and apoptosis were partially reversed after using 2-DG pretreatment to block the glycolysis pathway (Figure 5). These results suggest that DAC induces ROS production and mediates apoptosis in leukemic cells partly due to the increase in glycolysis flux.

Many clinical studies have shown that DAC has a killing effect on CML cells [26, 27]. BCR-ABL is a common fusion gene in hematopoietic malignancies and is present in approximately 20% of adult acute lymphoblastic leukemia and in more than 95% of CMLs. BCR-ABL can activate a variety of transcription factors, and the most important among these are STAT1 and STAT5. These two transcription factors are continuously activated in BCR-ABL-positive cell lines and primary cells isolated from CML patients, which can independently grow cytokines [28]. In normal cells, STATs enter the nucleus only when cytokines bind to receptors and activate JAK kinase. However, in CML, STATs can be directly activated by binding with tyrosine residues phosphorylated on BCR-ABL through its SH2 structure, and it appears that this does not need to rely on JAK activation [29]. To some extent, the activated STAT5 can prevent programmed cell death by upregulating antiapoptotic factor BCL-xL and AKT inhibiting proapoptotic factor BAD [30]. The present study revealed that DAC-induced apoptosis in K562/R and K562/TIGAR cells was stronger than imatinib and when compared with K562 cells (Figure 5(b)). This suggests that DAC can also promote the apoptosis of myeloid leukemia cells by downregulating the expression of BCR-ABL. Therefore, DAC therapy may improve the prognosis of patients with CML in blast crisis.

Autophagy is an evolutionarily conservative mechanism for the degradation of intracellular substances, which is responsible for the circulation of metabolites and the maintenance of intracellular stability [31]. In particular, TIGAR has the ability to limit the autophagy activity of cancer cells by regulating the production of ROS. Jia-Ming et al. reported that TIGAR has dual effects on the survival of cancer cells through the inhibition of apoptosis and autophagy [32]. Ting et al. reported that the knockdown of TIGAR induces the apoptosis and autophagy formation of breast cancer cells by increasing the intracellular ROS level, thereby enhancing the antitumor effect of physapubenolide [33]. As the upstream signaling pathway of autophagy, the molecular regulatory mechanism of ROS mainly includes transcriptional regulation and posttranscriptional regulation. High levels of ROS stimulate the activity of P53, HIF-1, NRF2, and FOXO3 in the nucleus, respectively, and enhances the transcription of the corresponding genes TIGAR/DRAM, BNIP3/NIX, p62, and LC3/BNIP3. In addition, ROS can also regulate PERK, and its downstream effectors regulate the transcription of autophagy-related genes [34]. In most chemotherapy regimens, the autophagy activity increased in varying degrees after treatment [35]. In agreement with other investigators, the DAC treatment induced the autophagy activation in HL-60 cells, and the knockdown of TIGAR significantly enhanced the DAC-induced autophagy (Figure 7). Autophagy is often considered to prevent cancer from developing. On the contrary, once cancer occurs, the increase in autophagy flux tends to enable tumor cells to survive and grow [36, 37]. Thus, autophagy activation induced by chemotherapeutic drugs can be considered as a strategy to protect tumor cells from being completely killed by the drugs.

## 5. Conclusions

Taken together, the present study revealed that in addition to hypomethylation, DAC can also play an antitumor role in myeloid leukemia by downregulating the TIGAR and BCR-ABL proteins. Autophagy is a self-protective mechanism of cancer cells after chemotherapy. However, it remains to be determined whether the inhibition of autophagy can further improve the apoptosis rate of DAC combined with TIGAR knockout in cells. Considering the dual regulatory effects of TIGAR on apoptosis and autophagy, TIGAR may be one of the drug targets for the clinical treatment of leukemia.

## Data Availability

The data used to support the findings of this study are available from the corresponding authors upon request.

## Supplementary Materials

Supplementary 1 Figure S1: DAC inhibits the TIGAR mRNA and protein expression. The TIGAR mRNA (a) and protein (b) levels were observed when K562 cells were treated with different concentrations of DAC (0.25, 0.50, and 1.00 *μ*M) for 24 hours. The TIGAR mRNA (c) and protein (d) levels were observed when K562 cells were treated with 0.5 *μ*M of DAC for different lengths of time (1, 3, 7, and 24 hours). ^∗^*P* < 0.05, ^∗∗^*P* < 0.01, ^∗∗∗^*P* < 0.001, NS *P* > 0.05 compared to the control group.

Supplementary 2 Table S1 Primer sequence used for qPCR.
