# Supplementary figures and images for: Decitabine Downregulates TIGAR to Induce Apoptosis and Autophagy in Myeloid Leukemia Cells
Source: Oxid Med Cell Longev. 2021 Jan 18;2021:8877460. doi: 10.1155/2021/8877460 (PMC7836025; doi:10.1155/2021/8877460)

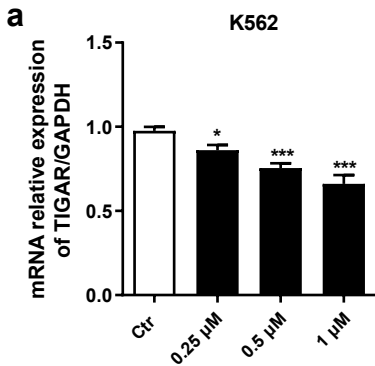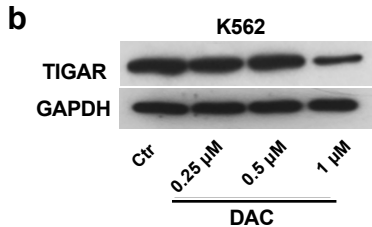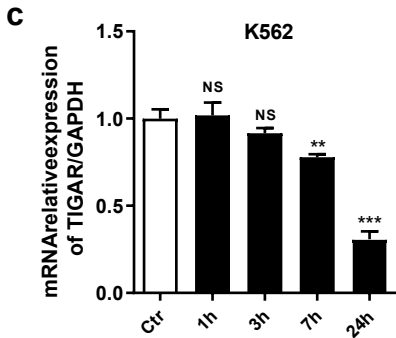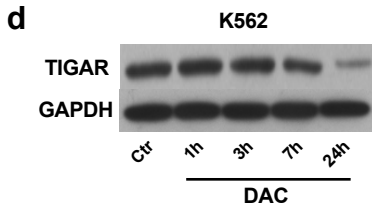

Supplement: Supplementary 1 — Figure S1: DAC inhibits the TIGAR mRNA and protein expression. The TIGAR mRNA (a) and protein (b) levels were observed when K562 cells were treated with different concentrations of DAC (0.25, 0.50, and 1.00 μM) for 24 hours. The TIGAR mRNA (c) and protein (d) levels were observed when K562 cells were treated with 0.5 μM of DAC for different lengths of time (1, 3, 7, and 24 hours). ∗P < 0.05, ∗∗P < 0.01, ∗∗∗P < 0.001, NS P > 0.05 compared to the control group. [file 8877460.f1.pdf]
