# Supplementary material for: Decitabine Downregulates TIGAR to Induce Apoptosis and Autophagy in Myeloid Leukemia Cells
Source: Oxid Med Cell Longev. 2021 Jan 18;2021:8877460. doi: 10.1155/2021/8877460 (PMC7836025; doi:10.1155/2021/8877460)
Supplement: Supplementary 2 — Table S1 Primer sequence used for qPCR. [file 8877460.f2.docx]

**Table S1** Primer sequence used for qPCR.

| **Gene name** | **Forward sequence** | **Reverse sequence** |
| --- | --- | --- |
| TIGAR | 5’- TAAAGTTAATTCAGACAGCGGT-3’ | 5’- GCTGGTAAGGAACACTTAAGGT-3’ |
| LC3 | 5’- GAACGATACAAGGGTGAGAAGCA-3’ | 5’- TGAGATTGGTGTGGAGACGCT-3’ |
| Beclin1 | 5’- ATCTGGCACAGTGGACAGTTTG-3’ | 5’- CCGTAAGGAACAAGTCGGTATC-3’ |
| ATG5 | 5’- GGATTTCGTTATATCCCCTTTA-3’ | 5’- ATCTCCTAGTGTGTGCAACTGT-3’ |
| p62 | 5’- GACCCGTCTACAGGTGAACTC-3’ | 5’- ACAGCATCTGGGAGAGGGACT-3’ |
| ATG3 | 5’- CACCACCTCCCATGTGTTCA-3’ | 5’-CGTTAACAGCCATTTTGCCACT-3’ |
| GAPDH | 5’-CCTCTGACTTCAACAGCGACAC-3’ | 5’-CTGTTGCTGTAGCCAAATTCGT-3’ |
